# Higher cord blood 25-hydroxyvitamin D concentrations reduce the risk of early childhood eczema: in children with a family history of allergic disease

**DOI:** 10.1186/s40413-015-0077-9

**Published:** 2015-10-06

**Authors:** Debra Jane Palmer, Thomas R. Sullivan, Clark M. Skeaff, Lisa G. Smithers, Maria Makrides, on behalf of the DOMInO Allergy Follow-up Team

**Affiliations:** Women’s & Children’s Health Research Institute, University of Adelaide, North Adelaide, SA 5006 Australia; School of Paediatrics and Child Health, University of Western Australia, Subiaco, WA 6008 Australia; School of Population Health, University of Adelaide, Adelaide, SA 5005 Australia; Department of Human Nutrition, University of Otago, Dunedin, 9054 New Zealand; Healthy Mothers, Babies and Children, South Australian Health and Medical Research Institute, Adelaide, SA 5000 Australia; School of Paediatrics and Reproductive Health, University of Adelaide, Adelaide, SA 5005 Australia

**Keywords:** Allergy prevention, Cord blood, Eczema, Pregnancy, Vitamin D

## Abstract

**Background:**

In recent years the role of vitamin D status in early life on the development of allergic disease has generated much interest. The aim of this study was to determine whether cord blood vitamin D concentrations were associated with risk of early childhood allergic disease.

**Methods:**

Measurements of cord blood 25-hydroxyvitamin D [25(OH)D] concentrations were available in 270 mother-child pairs who were participating in the allergy follow-up (*n* = 706) of the Docosahexaenoic Acid to Optimise Mother Infant Outcome randomised controlled trial. All of the children had a hereditary risk of allergic disease. The diagnosis of allergic disease was made during medical assessments at 1 and 3 years of age.

**Results:**

The mean (standard deviation) standardised cord blood 25(OH)D concentration was 57.0 (24.1) nmol/L. The cumulative incidence of eczema to 3 years of age, *n* = 101/250 (40 %) was associated with standardised cord blood 25(OH)D concentration, with a 10 nmol/L rise in 25(OH)D concentration reducing the risk of eczema by 8 % (relative risk 0.92, 95 % confidence interval 0.86–0.97; *P* = 0.005). This association was stronger at 1 year of age, when a 10 nmol/L rise in standardised cord blood 25(OH)D concentration reduced the risk of eczema by 12 % (relative risk 0.88, 95 % confidence interval 0.81–0.96; *P* = 0.002). No associations between cord blood 25(OH)D concentrations and development of allergic sensitisation, allergic rhinitis or asthma in early childhood were found.

**Conclusion:**

In children with a family history of allergic disease, a higher cord blood 25(OH)D concentration appears to be associated with reduced risk of eczema in early childhood.

**Trial registration:**

Australian New Zealand Clinical Trials Registry ACTRN12610000735055 (DOMInO trial: ACTRN12605000569606).

## Background

Vitamin D is well known for its role in bone metabolism, however in recent years, the effects of vitamin D on immune function and the development of allergic disease has generated much interest. Some epidemiological studies have shown that lower maternal vitamin D dietary intakes during pregnancy increases the risk of allergic disease; atopic dermatitis/eczema [1], food allergen sensitisation [2], asthma [3] and allergic rhinitis [3] in the offspring. Studies to date investigating the associations between objective measures of maternal vitamin D status during pregnancy and allergic disease outcomes in childhood have been limited. Three recent studies [4–6] have found that higher cord blood vitamin D concentrations were protective against the development of early childhood atopic dermatitis/eczema, respiratory tract infection and wheezing, but not asthma or allergic rhinitis. However childhood allergy outcomes in these three studies [4–6] relied on parent reported history of doctor diagnosed outcomes rather than direct standardised medical practitioner clinical assessments.

The best indicator of vitamin D status is considered to be the measurement of serum 25-hydroxyvitamin D (25(OH)D), which is the most abundant and stable vitamin D metabolite [7]. Serum 25(OH)D concentrations reflect vitamin D produced from sunlight exposure and intake from foods and supplements. It has been well established that serum 25(OH)D concentrations ≥ 50 nmol/L facilitate optimal bone health (calcitropic function of vitamin D) [8]. However in recent years there has been much discussion about the ideal serum 25(OH)D concentrations needed for other health outcomes, with suggestions [9] that concentrations ≥ 75 nmol/L may be needed for optimal immune function and for reducing the risk of allergic disease. During pregnancy, the fetus is exposed to vitamin D through the cord blood supply and the ability of 25(OH)D to cross the placenta [10]. Cord blood 25(OH)D concentrations are highly correlated with infant [11, 12] and maternal serum 25(OH)D concentrations [11, 12], although they are usually lower compared with maternal concentrations [13–15].

This paper reports the associations between cord blood 25(OH)D concentrations and standardised clinically assessed allergic disease outcomes to 3 years of age in children with a family history of allergic disease, whose mothers participated in a double-blinded, multi-centre randomised controlled trial of omega-3 long-chain polyunsaturated fatty acids supplementation in pregnancy [16–18].

## Methods

### Subjects and study design

Children (*n* = 706) who had a mother, father or sibling with a history of medically diagnosed allergic disease, and whose mothers were participants in the Docosahexaenoic Acid (DHA) to Optimise Mother Infant Outcome (DOMInO) Trial [16], participated in the allergy follow-up study of the DOMInO Trial [17, 18]. Briefly, in the DOMInO trial women allocated to the omega-3 long chain polyunsaturated fatty acid group were asked to consume three 500 mg capsules of fish oil concentrate, providing 800 mg of DHA and 100 mg of eicosapentaenoic acid (EPA). Women in the control group were asked to take three 500 mg vegetable oil capsules without omega-3 long chain polyunsaturated fatty acids daily. Neither of these capsules (intervention or control group) contained vitamin D. Women took capsules from 21 weeks’ gestation until delivery. Among the 706 child participants in the allergy follow-up study, measurements of cord blood 25(OH)D concentrations were available in 270 mother-child pairs. Approval for this study was granted by the Human Research Ethics Committees of the Women’s and Children’s Hospital and Flinders Medical Centre, Adelaide, Australia.

### Measurement of cord blood 25(OH)D concentration

Samples of umbilical cord blood were collected at birth. Plasma was separated from whole blood by centrifugation and immediately stored at −20 °C. Samples were shipped to the Steroid and Immunobiochemistry Laboratory (Christchurch, New Zealand) on dry ice. Analysis of 25(OH)D was conducted using a liquid chromatography-tandem mass spectroscopy (LC-MS/MS) method [19]. Accuracy of the method was assessed using Standard Reference Material® 972 from the National Institute of Standards and Technology (NIST). We determined 25(OH)D3 concentrations in levels 1, 2, and 3 of the Standard Reference Material® 972 to be 60 nmo/L, 30 nmo/L, and 47 nmol/L, respectively, compared with the NIST Certified Concentration Values of 60 nmol/L, 31 nmol/L and 46 nmol/L, respectively. The precision of the method was 6–7 % and was established by repeat (*n* = 16) measurements of three control serum samples 25(OH)D status with concentrations of 23 nmol/L, 59 nmol/L and 107 nmol/L. 25(OH)D concentrations are presented as nmol/L.

### Early childhood allergic disease outcome assessments and definitions

Children participating in the allergy follow-up of the DOMInO Trial attended a medical review appointment at 1 and 3 years of age [17, 18]. Allergic sensitisation was defined as a positive skin prick test (weal ≥3 mm above negative control) to at least one of the food allergens (hens’ egg, cows’ milk, wheat, tuna, peanut, cashew nut and sesame seed) or aeroallergens (ryegrass pollen, olive tree pollen, *Alternaria tenuis*, cat hair and house dust mites -*Dermatophagoides pteronyssinus* and *Dermatophagoides farinae*) assessed. Eczema was defined as the presence of eczema, criteria according to Hanifin and Rajka [20], on medical review or a history of an itchy rash distributed to the facial, flexural, or extensor surface of the skin that had followed a fluctuating or chronic course. IgE-associated food allergy was defined as a history of ingestion of a food with immediate reaction (< 60 min), including skin rash (hives, rash, or swelling) with or without respiratory symptoms (cough, wheeze, stridor), gastrointestinal symptoms (abdominal pain, vomiting, loose stools), or cardiovascular symptoms (collapse), coupled with sensitisation to the implicated food. Asthma was defined as a history of 3 or more episodes of wheeze with the episodes less than 6 weeks apart and/or daily use of asthma medication. Allergic rhinitis was defined as a history of sneezing, or a runny, or blocked nose accompanied by itchy-watery eyes when there have not been symptoms to suggest an upper respiratory tract infection. Respiratory tract infections were defined as a parent reported history of medically diagnosed bronchiolitis or Respiratory Syncytial Virus.

### Statistical methods

25(OH)D concentrations were standardised to account for variability in cord blood 25(OH)D by month of birth. Firstly, an overall weighted mean 25(OH)D concentration was calculated by weighting individual measurements by the inverse of the total number of measurements in the corresponding month (to account for variation in the number of observations in different months). Secondly, the residuals from a regression model of 25(OH)D on month (treated as a categorical variable) were added to the overall mean 25(OH)D concentration to create standardised 25(OH)D concentrations. A similar approach was used by Jenab et al. [21].

For all allergy outcomes, log binomial regression models were used to estimate the effect of a 10 nmol/L increase in standardised 25(OH)D on the risk of allergy. All associations were described using relative risks with 95 % confidence intervals. Statistical models were adjusted for the following potential confounders; DHA treatment group, parity, sex and maternal smoking during or leading up to pregnancy. The confounders were determined *a priori* based on previous research showing they are commonly associated with the development of atopic disease [22]. To avoid over-fitting statistical models to outcomes with relatively low incidence, only a limited number of potential confounders were considered prior to analysis. For outcomes where it was not possible to achieve model convergence with adjustment for covariates, results of unadjusted models are presented. Since data were obtained from a randomised trial, effect modification of 25(OH)D by DHA treatment group was also investigated by adding interaction effects to statistical models. Checks were performed to ensure adequate model fit throughout, with modified Hosmer-Lemeshow tests suggesting that the inclusion of standardised 25(OH)D as a continuous predictor was reasonable for all the allergy outcomes considered. Statistical significance was assessed at the two sided *P* < 0.05 level. All analyses were performed using SAS version 9.3.

## Results

The sample of 270 mother-child pairs with available measurements of cord blood 25(OH)D concentrations were representative of the overall allergy follow-up study population (see Table 1).

**Table 1:** Comparison of baseline characteristics between those with cord blood vitamin D (25 (OH) D) concentration results and total allergy follow-up cohort

Characteristic	Vitamin D Cohort (*n* = 270)	Total Cohort (*n* = 706)
Mother’s age at trial entry (years) ^a^	28.8 (5.6)	29.5 (5.7)
Race – Caucasian	256 (94.8)	672 (95.2)
Mother completed secondary education	171 (63.3)	454 (64.3)
Mother smoked during or leading up to pregnancy	73 (27.0)	205 (29.0)
Primiparous	110 (40.7)	281 (39.8)
Maternal history of allergic disease	182 (67.4)	493 (69.8)
Dual parental history of allergic disease	72 (26.7)	206 (29.2)
Infant sex (male)	129 (47.8)	337 (47.7)
Treatment group		
DHA	130 (48.1)	368 (52.1)
Control	140 (51.9)	338 (47.9)

Values are numbers (percentages) unless indicated otherwise

*DHA* Docosahexaenoic acid

^a^ Mean (± SD)

### Vitamin D

**Fig. 1:**
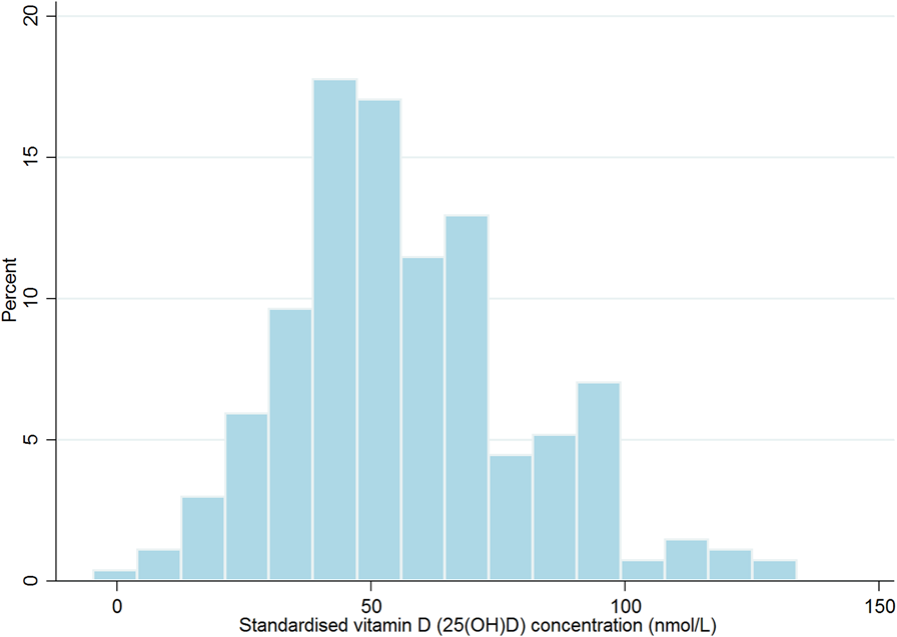
The distribution of standardised cord blood 25(OH)D concentrations

The mean (SD) raw cord blood 25(OH)D concentration was 55.9 (28.4) nmol/L and the mean (SD) standardised 25(OH)D concentration was 57.0 (24.1) nmol/L. The distribution of cord blood standardised 25(OH)D concentrations are displayed in Fig. 1. Characteristics of the participants and standardised 25(OH)D concentrations are shown in Table 2. Standardised 25(OH)D concentrations were higher for Caucasian mothers (*n* = 256) with mean (SD) of 58.1 (24.1) nmol/L compared to non-Caucasian mothers (*n* = 14) with mean (SD) of 37.8 (14.7) nmol/L, *P* = 0.002. For other participant characteristics that may potentially play a role in allergic disease development, DHA supplementation, maternal smoking, parity and infant sex, the standardised 25(OH)D concentrations did not differ between groups.

**Table 2:** Characteristics of participants and standardised vitamin D (25OH) D) concentrations

Characteristic	25 (OH) D nmol/L^a^	N	<25 nmol/L	25–49.9 nmol/L	50–74.9 nmol/L	≥75 nmol/L
Total cohort	57.0 ± 24.1	270 (100)	19 (7.0)	97 (35.9)	103 (38.2)	51 (18.9)
Treatment group						
Control	56.9 ± 24.5	140 (51.9)	11 (7.9)	47 (33.6)	55 (39.3)	27 (19.3)
DHA	57.2 ± 23.8	130 (48.1)	8 (6.2)	50 (38.5)	48 (36.9)	24 (18.5)
Mother smoked during or leading up to pregnancy						
Yes	54.7 ± 21.7	73 (27.0)	6 (8.2)	28 (38.9)	28 (38.9)	11 (15.1)
No	57.9 ± 24.9	197 (73.0)	13 (6.6)	69 (35.0)	75 (38.1)	40 (20.3)
Parity						
0	55.4 ± 25.7	110 (40.7)	11 (10.0)	40 (36.4)	39 (35.5)	20 (18.2)
> = 1	58.2 ± 23.0	160 (59.3)	8 (5.0)	57 (35.6)	64 (40.0)	31 (19.4)
Infant sex						
Male	59.0 ± 26.0	129 (47.8)	7 (5.4)	44 (34.1)	52 (40.3)	26 (20.2)
Female	55.2 ± 22.2	141 (52.2)	12 (8.5)	53 (37.6)	51 (36.2)	25 (17.7)

Values are numbers (percentages) unless indicated otherwise

*DHA* Docosahexaenoic acid

^a^ Mean ± SD all such values

### Early childhood allergic disease outcomes

Overall the cumulative incidence of any allergic disease (eczema, IgE-mediated food allergy, allergic rhinitis and/or asthma) by 3 years of age was 57 % (137/240) in this cohort (with a family history of allergic disease). Consistent with the usual pattern of expression of the allergic phenotype, eczema was the most common allergic disease in the first three years of life [23], with a cumulative incidence of 40 % (101/250).

There was no evidence for effect measure modification by DHA treatment group in any of the allergic disease outcomes, so interaction effects were not included in the final models. The risk of eczema at 1 year of age decreased as cord blood 25(OH)D concentration increased; a 10 nmol/L rise in standardised cord blood 25(OH)D concentration was associated with a 12 % reduction in risk (relative risk 0.88, 95 % confidence interval 0.81–0.96; *P* = 0.002 (Table 3). The cumulative incidence of eczema to 3 years of age was also associated with cord blood 25(OH)D concentration, with a 10 nmol/L rise in standardised cord blood 25(OH)D concentration reducing the risk by 8 % (relative risk 0.92, 95 % confidence interval 0.86–0.97; *P* = 0.005). A similar relationship was observed for eczema at 3 years of age, however the association did not reach statistical significance (Table 3).

**Table 3:** Associations between standardised vitamin D (25 (OH) D) levels and allergic disease outcomes

Allergic disease outcome	Incidence^a^	Unadjusted RR^b^ (95 % CI)	Adjusted RR^c^ (95 % CI)	Adjusted^c^
				*P* Value
Eczema at 1 year of age	70/265 (26.4)	0.89 (0.81, 0.97)	0.88 (0.81, 0.96)	0.002
Eczema with sensitisation at 1 year of age	19/265 (7.2)	0.85 (0.70, 1.04)	0.84 (0.69, 1.01)	0.07
Eczema at 3 years of age	77/247 (31.2)	0.94 (0.87, 1.01)	0.93 (0.86, 1.01)	0.07
Eczema with sensitisation at 3 years of age	26/244 (10.7)	0.87 (0.74, 1.02)	0.85 (0.73, 1.00)	0.051
Cumulative incidence of eczema by 3 years of age	101/250 (40.4)	0.92 (0.86, 0.98)	0.92 (0.86, 0.97)	0.005
Cumulative incidence of eczema with sensitisation by 3 years of age	30/243 (12.3)	0.85 (0.73, 0.98)	0.83 (0.72, 0.96)	0.01
IgE-mediated food allergy at 1 year of age	4/260 (1.5)	0.60 (0.41, 0.90)	-	0.01^d^
IgE-mediated food allergy at 3 years of age	7/229 (3.1)	0.78 (0.54, 1.13)	0.77 (0.50, 1.17)	0.22
Cumulative incidence IgE-mediated food allergy by 3 years of age	8/228 (3.5)	0.80 (0.57, 1.12)	0.78 (0.55, 1.11)	0.17
Any sensitisation at 1 year of age	36/260 (13.8)	0.95 (0.84, 1.09)	0.94 (0.83, 1.07)	0.36
Any sensitisation at 3 years of age	52/229 (22.7)	0.96 (0.87, 1.07)	0.95 (0.86, 1.05)	0.28
Cumulative incidence any sensitisation by 3 years of age	66/230 (28.7)	0.96 (0.88, 1.05)	0.95 (0.87, 1.03)	0.23
Cumulative incidence of allergic rhinitis by 3 years of age	39/246 (15.9)	0.97 (0.86, 1.10)	0.98 (0.87, 1.11)	0.80
Cumulative incidence of allergic rhinitis with sensitisation by 3 years of age	13/245 (5.3)	0.95 (0.75, 1.19)	0.94 (0.75, 1.18)	0.60
Cumulative incidence of asthma by 3 years of age	32/248 (12.9)	1.03 (0.91, 1.17)	1.03 (0.90, 1.18)	0.67
Cumulative incidence of asthma with sensitisation by 3 years of age	5/245 (2.0)	1.05 (0.74, 1.48)	-	0.80^d^
Cumulative incidence of any allergic disease by 3 years of age	137/240 (57.1)	0.95 (0.90, 0.99)	0.95 (0.90, 0.99)	0.02
Cumulative incidence of any allergic disease with sensitisation by 3 years of age	40/228 (17.5)	0.88 (0.78, 1.00)	0.87 (0.77, 0.98)	0.03
Respiratory tract infections by 1 year of age	45/267 (16.9)	1.08 (0.98, 1.20)	1.07 (0.97, 1.18)	0.18
Cumulative incidence of respiratory tract infections by 3 years of age	70/250 (28.0)	1.03 (0.95, 1.11)	1.02 (0.95, 1.10)	0.61

*RR* Relative risk

^a^ Values are numbers (percentages)

^b^ Relative Risk of outcome corresponding to a 10 unit increase in standardised 25(OH)D

^c^ Adjusted for treatment group, parity, gender and maternal smoking during or leading up to pregnancy

^d^ Unadjusted p-value reported due to low incidence of outcome

The cumulative incidence of eczema with sensitisation by 3 years of age was also associated with cord blood 25(OH)D concentration, with a 10 nmol/L rise in standardised cord blood 25(OH)D concentration reducing the risk by 17 % (relative risk 0.83, 95 % confidence interval 0.72–0.96; *P* = 0.01). We also observed a reduction in the risk, which did not reach statistical significance, for eczema with sensitisation at 1 year of age and at 3 years of age (Table 3).

Medical diagnosis of IgE-mediated food allergy at 1 year of age was uncommon in this cohort, only being identified in 4/260 (1.5 %) children. The risk of IgE-mediated food allergy at 1 year of age (but not at 3 years of age) also decreased as cord blood 25(OH)D concentration increased (Table 3). No associations between cord blood 25(OH)D concentrations and risk of development of allergic sensitisation, allergic rhinitis, asthma or respiratory tract infections in early childhood were found (Table 3).

In exploratory analyses, we examined whether the effect of standardised vitamin D on the allergic disease outcomes was modified by mother’s smoking status, cat ownership or dog ownership. As the majority of the participants were breastfed and of Caucasian race, we did not examine effect modification by breastfeeding status or race. There was evidence that the effect of standardised vitamin D on the cumulative incidence of any sensitisation by 3 years of age was modified by mother’s smoking status (interaction *p*-value = 0.03). A 10 nmol/L increase in standardised cord blood 25(OH) concentration was associated with a 9 % reduction in the risk of any sensitisation at 1 or 3 years in children whose mother did not smoke during or leading up to pregnancy (*RR* = 0.91; 95 % CI 0.82, 1.00). In contrast, for children with a mother that did smoke, a 10 nmol/L increase in cord blood 25(OH)D concentration was associated with an 11 % increase in the risk of any sensitisation at 1 or 3 years (*RR* = 1.11; 95 % CI 0.96, 1.28). No evidence of effect modification was found for any other allergic disease outcomes.

## Discussion

Our results of a 10 nmol/L rise in standardised cord blood 25(OH)D concentration being associated with reduced risk of eczema are consistent with the findings of other observational studies, which have also reported that higher cord blood 25(OH)D concentrations are associated with decreased risk of eczema [4, 6]. There have also been recent studies [15, 24, 25] which have found no association with cord blood 25(OH)D concentration and eczema outcomes in early childhood. Interestingly, our study also found that the cumulative incidence of eczema with sensitisation by 3 years of age was also associated with cord blood 25(OH)D concentration. Previous other studies investigating cord blood 25(OH)D concentrations have not reported results for atopic eczema (eczema with confirmed sensitisation). Other studies have reported food and/or inhalant allergen sensitisation independent of allergic disease outcomes and in accordance with our findings most [6, 24, 25] have found no association of cord blood 25(OH)D concentration with child sensitisation status. However Rothers et al. [26] in the United States identified a U-shaped relationship with lower (< 50 nmol/L) and higher (> 100 nmol/L) cord blood 25(OH)D concentrations associated with increased inhalant allergen sensitisation.

There are several biological pathways which may account for a beneficial effect of vitamin D in reducing eczema risk. The pathogenesis of eczema involves immune dysregulation, altered epidermal barrier function and inadequate bacterial defence [27] and vitamin D is known to have a regulatory influence on all of these [28]. Thus, vitamin D deficiency in early life is a strong candidate for predisposition to eczema. These biological effects are in keeping with observational studies that have indicated a link between vitamin D status and eczema outcomes, including lower serum vitamin D concentrations associated with increased incidence and severity of eczema symptoms [29].

We also found that higher cord blood 25(OH)D concentrations were associated with decreased risk of development of IgE-mediated food allergy in infancy. However this result should be interpreted with caution due to the low incidence of medically diagnosed IgE-mediated food allergy at 1 year of age (1.5 %) in our cohort. Our incidence was substantially lower than that reported in another recent Australian cohort, where food challenges identified > 10 % of 1 year olds to have IgE-mediated food allergy [30]. The inability to perform food challenges (due to insufficient resources) may explain the lower incidence of IgE-mediated food allergy at 1 year of age in our cohort. In contrast, a cohort study by Weisse et al. [15] found higher maternal blood (34 weeks gestation) as well as cord blood 25(OH)D concentrations to be associated with increased risk of parent reported doctor diagnosed food allergy, with an incidence of 5.5 % by 2 years of age. Whereas other studies [6, 25] have found no association of cord blood 25(OH)D concentrations with the development of food allergy. Interestingly, the Weisse et al. [15] German cohort study was conducted at a higher latitude of 51^0^ N (compared to our study at 35^0^S) with lower median (inter-quartile range) cord blood concentration of 27 nmol/L (17–43 nmol/L). These lower average cord blood concentrations may have contributed to their contrasting finding with regard to food allergy outcomes and the finding of no association with offspring eczema outcomes.

The pattern of allergic disease is known to differ with age, with the greatest incidence of food allergy and atopic dermatitis/eczema being in the first few years of life, while asthma and allergic rhinitis continue to rise until adulthood. Consistent with the recent findings of other cohort studies, we found no association of cord blood 25(OH)D concentrations with asthma [4, 5, 24, 26] or allergic rhinitis [4, 5, 24, 26] outcomes. We acknowledge that longer term follow-up of these children is needed before this result can be conclusive and we are currently following up the children in our cohort at 6 years of age for allergic disease outcomes.

The strengths of our study include standardised medical assessments of allergic disease outcomes, with high follow up rates of > 95 % at 1 year and > 90 % at 3 years of age. We also calculated and reported standardised cord blood 25(OH)D concentrations for our results, which is important to adjust for seasonal variability in 25(OH)D concentrations due to month of birth. We acknowledge that a limitation of our study was that we have not taken blood samples from the children to examine vitamin D status at multiple time-points during early life. It may be that the timing of differential vitamin D status could have variable effects on the development of allergic diseases. A family history of allergic disease in all the participants in our study may reduce the ability to generalize our findings to the whole population, however Biaz et al. [4] who did not select participants based on a family history of allergic disease also reported that higher cord blood 25(OH)D concentrations were associated with decreased risk of early childhood eczema. Another possible limitation to the generalisability of our results was that we had a predominance of Caucasian mothers (95 %). Since Caucasian mothers were found to have higher cord blood 25(OH)D concentrations compared to non-Caucasian mothers, replication of our investigations would be beneficial in study populations with a predominance of non-Caucasian participants.

There are several randomised controlled trials currently investigating the effect of maternal vitamin D supplementation during pregnancy on allergic disease outcomes (NCT00920621 and NCT00856947). The level of supplementation tested in these trials should be adequate to attain a significant shift in maternal 25(OH)D levels and therefore provide definitive evidence regarding the effect of vitamin D during pregnancy on early childhood allergic disease.

## Conclusion

In children with a family history of allergic disease, higher cord blood 25(OH)D concentrations appear to be associated with a reduction in the risk of eczema development through to 3 years of age.

## Abbreviations

25(OH)D: 25-hydroxyvitamin D
DHA: Docosahexaenoic Acid
DOMInO: Docosahexaenoic acid to Optimise Mother Infant Outcome
IgE: Immunoglobulin E
